# Fast Forward Science: Risks and Benefits in the Rapid Science of COVID-19

**DOI:** 10.1007/978-3-030-65355-2_31

**Published:** 2021-03-20

**Authors:** Jelte Wicherts

**Affiliations:** 1 Tilburg University, Tilburg, The Netherlands; 2 Tilburg University, Tilburg, The Netherlands; 3 Tilburg University, Tilburg, The Netherlands; 4 Tilburg University, Tilburg, The Netherlands; Department of Methodology and Statistics, Tilburg School of Social and Behavioral Sciences, Tilburg, The Netherlands

## Abstract

Since the onset of the SARS-COV-2 pandemic in late 2019, the scientific literature on the SARS-COV-2 virus and the disease COVID-19 has a growth rate that resembles the growth in confirmed COVID-19 cases that continue to make media headlines all across the globe. Biomedical coronavirus research started slowly but increased to hundreds of articles per week—not unlike the spread of the virus itself. At the time of writing in mid-2020, around 2500 publications per week appear in PubMed on COVID-19 or SARS-COV-2. This new biomedical literature has emerged at an unprecedented but will the scientific community be able to end the suffering caused by the pandemic? Can we trust the insights from the rapidly emerging scientific literature on the coronavirus to implement wide-ranging social, economic, and health policies and vaccination programs? To answer these questions, I here relate the rapid science on the coronavirus pandemic to regular biomedical science and the meta-scientific insights on it. I focus my attention on peer reviews, open access, retractions, open data, and registration of studies.

Since the onset of the SARS-COV-2 pandemic in late 2019, the scientific literature on the SARS-COV-2 virus and the disease COVID-19 has a growth rate that resembles the growth in confirmed COVID-19 cases that continue to make media headlines all across the globe. Figure 31.1 displays the number of publications listed in the scholarly publication platform PubMed that can be found with the string “COVID-19 OR SARS-COV-2” for all 26 weeks representing the first half of 2020. It shows that biomedical coronavirus research started slowly but increased to hundreds of articles per week—not unlike the spread of the virus itself. At the time of writing in mid-2020, around 2500 publications per week appear in PubMed on COVID-19 or SARS-COV-2. The curve appears to be flattening but we need to keep in mind the delay in posting of records in PubMed. The actual scientific literature on the coronavirus is even bigger because PubMed is restricted to biomedical outlets and does not cover the many other scientific fields that help us better understand and deal with the pandemic. This new biomedical literature has emerged at an unprecedented pace and highlights the commitment of thousands of researchers all over the globe to understand the virus and its spread, to develop a vaccine, to find treatments for those afflicted, and to ultimately end the pandemic suffering.

**Fig. 31.1:**
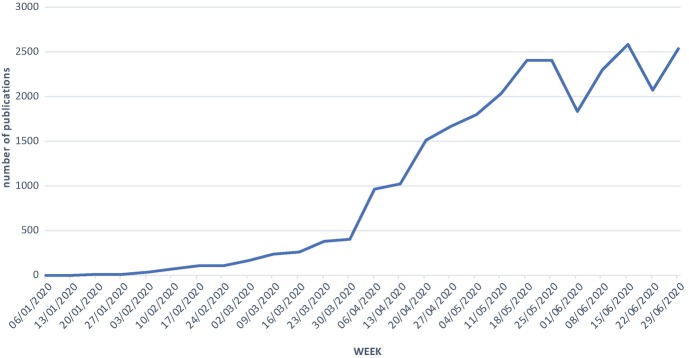
Growth of the COVID-19/SARS-COV2 literature in the first half of 2020 in PubMed (source: author)

Will the scientific community be able to end the suffering caused by the pandemic? Can we trust the insights from the rapidly emerging scientific literature on the coronavirus to implement wide-ranging social, economic, and health policies and vaccination programs? To answer these questions, I here relate the rapid science on the coronavirus pandemic to regular biomedical science and the meta-scientific insights on it. I focus my attention on peer reviews, open access, retractions, open data, and registration of studies. I end with an optimistic conclusion.

## Rapid Peer Review

The vast pace in publishing in the literature on the coronavirus reflects the speed of setting up studies, conducting the research, analyzing outcomes, writing up results, and the peer review process that seeks to independently check the quality of the work. With respect to the latter, we know that the typical review process at biomedical journals takes 3–4 months. For the early articles reporting coronavirus research, the median publication lag was 11 days (Kun 2020). This begs the question of how well reviewers are able to critically assess the quality of the work under such intense time pressure.

## Open Access

One way to deal with limitations of the closed system of pre-publication peer review is to increase the number of critical readers by publishing work without any restrictions under open access. In the first half of 2020, the coronavirus literature included 27,373 publications in PubMed. In the same period, the literatures on cancer and cardiovascular diseases—the leading causes of death in the Western world—included 95,527 and 30,728 publications, respectively. Two-thirds of the coronavirus publications (18,715 or 68%) are publicly available under open access. These percentages are markedly lower in cancer research (42,775 or 45%) and cardiovascular research (12,892 or 42%). Open access improves the dissemination of results and increases the number of potential post-publication reviewers by the thousands. In this sense, the biomedical literature on the coronavirus is more open than ever.

## Errors and Retractions

A main corrective mechanism of science is to avoid the publication of sloppy research through peer review. But if sloppy research gets published after having passed peer review anyway, we can only hope that attentive readers scrutinize the publication and correct the record by publishing critiques or by corresponding to the editor that something in the original publication does not smell right. In that case, the editor might choose to retract the publication altogether. In that respect, retractions might reflect the self-corrective mechanism of a field. Interestingly, the retraction rate of coronavirus publications is markedly higher than that in the wider literature (Yeo-Teh and Tang 2020). Surely, any retraction highlights a problem but also indicates that readers took action to correct the literature.

## Open Data

Trust in scientific findings can be enhanced by sharing the data underlying studies, allowing others to scrutinize the results through reanalyzes. Open data also allows many more researchers to work with the data. In the open science era, we see an enormous growth in open data sets and open resources. This is not different for coronavirus research anno 2020; in their review of open data resources relating to the coronavirus, Alamo et al. (2020) listed no fewer than 152 links to websites housing open data or data resources that can be used to study the coronavirus. Surely, even today, there are still influential studies being published that fail to share data, but such obscurity will increasingly become obsolete if funders, researchers, editors, and publishers really want to present the best research that can withstand any scrutiny. Open science strengthens truth finding.

## Registrations

**Fig. 31.2:**
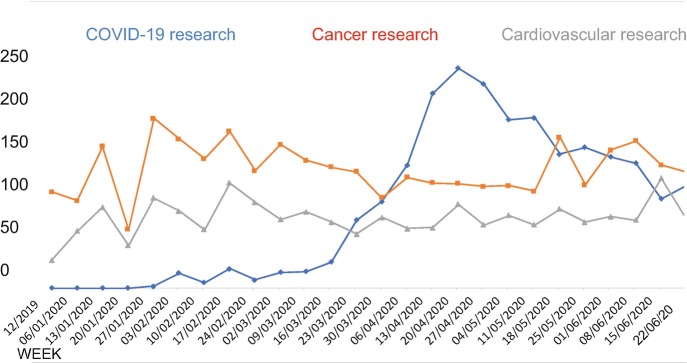
Registered studies clinicaltrials.gov (source: author)

An earmark of methodological rigor that helps avoid selective publication of results based on their outcomes (publication bias) and counters many other biases in the analysis and reporting of research results is the registration of studies prior to data collection. Most randomized clinical trials are nowadays registered via platforms such as clinicaltrials.gov, if only because major medical journals would simply not consider publishing an unregistered trial. A quick and easy search on Clinicaltrials.gov indicates that, in the first 6 months of 2020, no fewer than 2250 studies on the coronavirus have been registered. Many of these studies represent randomized controlled trials that test the efficacy of drugs to treat COVID-19 patients and early phase trials to study the working and safety of the much-desired vaccines that could end the pandemic. Figure 31.2 indicates the number of COVID-19 or SARS-COV-2 studies newly registered per week in this period. By comparison, there were 3355 new registrations for cancer research and 1974 for cardiovascular research in the same period. In other words, COVID-19 has become one of the main targets of biomedical science in a matter of 2 months! Over 100 studies are being registered per week on this platform, and the current 2000 studies are mostly still running.

## Light at the End of the Tunnel

Almost everyone in the world has been affected by social distancing policies due to the coronavirus pandemic, and a lot of people have suffered or passed away due to COVID-19. Many more will unfortunately perish because of it. But the graphs in Figs. 31.1 and 31.2 provide hope that the scientific community will beat the virus. This will not be an easy process. Scientific progress has never been a linear path up the mountain of knowledge. Scientific progress as we have seen it so many times in our history involved many dead ends, false positive findings, overhyped claims, dishonest science, wasted resources, biased analyses, fierce debates, erroneous methods, sloppy science, and the occasional major breakthrough. At the current rate, the literature on the coronavirus grows with over 350 publications per day. Many of these publications will later prove to be useless or flat out wrong. COVID-19 is an entirely new disease and hence research on it is expected to be noisy. It would be unrealistic to expect the emerging field to offer instantaneous results that are valid. Instead, we should expect the majority of findings to be false, biased, ignored, and later corrected by better designed and more rigorous studies. But we do not need all results of all studies to be definitive. We do not need 100% accuracy or 150 different vaccines for the same virus.

As long as scientists work transparently, sharing their work, data, and research plans online, and as long as scientists are overwhelmingly interested in the truth, science will go ahead and progress will be made. There is no way of telling when to expect the needed breakthroughs. Science is certainly not functioning optimally and could surely become more efficient. But science anno 2020 is bigger, faster, and more transparent than it ever was. The rapid science of COVID-19 and SARS-COV-2 is not perfect, but it offers hope and ultimately a solution to the coronavirus crisis. We might even expect the movement towards more rapid, open, self-corrective, and meticulous research to persist after the crisis to create a science that is more resistant to false claims and better equipped to promote global health and well-being.
